# Advancing wide implementation of precision oncology: A liquid nitrogen‐free snap freezer preserves molecular profiles of biological samples

**DOI:** 10.1002/cam4.5781

**Published:** 2023-03-14

**Authors:** Hanneke van der Wijngaart, Sahil Jagga, Henk Dekker, Richard de Goeij, Sander R. Piersma, Thang V. Pham, Jaco C. Knol, Babs M. Zonderhuis, Harry J. Holland, Connie R. Jiménez, Henk M. W. Verheul, Srinivas Vanapalli, Mariette Labots

**Affiliations:** ^1^ Department of Medical Oncology Amsterdam UMC Location Vrije Universiteit Amsterdam Amsterdam The Netherlands; ^2^ Applied Thermal Sciences, Faculty of Science and Technology University of Twente Enschede The Netherlands; ^3^ Cancer Center Amsterdam, Imaging and Biomarkers Amsterdam The Netherlands; ^4^ Department of Surgery Amsterdam UMC Location Vrije Universiteit Amsterdam Amsterdam The Netherlands; ^5^ Department of Medical Oncology Erasmus University Medical Center Rotterdam The Netherlands

**Keywords:** biopsies, cancer, cooling, multi‐omics, precision oncology, snap freezing

## Abstract

**Purpose:**

In precision oncology, tumor molecular profiles guide selection of therapy. Standardized snap freezing of tissue biospecimens is necessary to ensure reproducible, high‐quality samples that preserve tumor biology for adequate molecular profiling. Quenching in liquid nitrogen (LN_2_) is the golden standard method, but LN_2_ has several limitations. We developed a LN_2_‐independent snap freezer with adjustable cold sink temperature. To benchmark this device against the golden standard, we compared molecular profiles of biospecimens.

**Methods:**

Cancer cell lines and core needle normal tissue biopsies from five patients' liver resection specimens were used to compare mass spectrometry (MS)‐based global phosphoproteomic and RNA sequencing profiles and RNA integrity obtained by both freezing methods.

**Results:**

Unsupervised cluster analysis of phosphoproteomic and transcriptomic profiles of snap freezer versus LN_2_‐frozen K562 samples and liver biopsies showed no separation based on freezing method (with Pearson's *r* 0.96 (range 0.92–0.98) and >0.99 for K562 profiles, respectively), while samples with +2 h bench‐time formed a separate cluster. RNA integrity was also similar for both snap freezing methods. Molecular profiles of liver biopsies were clearly identified per individual patient regardless of the applied freezing method. Two to 25 s freezing time variations did not induce profiling differences in HCT116 samples.

**Conclusion:**

The novel snap freezer preserves high‐quality biospecimen and allows identification of individual patients' molecular profiles, while overcoming important limitations of the use of LN_2_. This snap freezer may provide a useful tool in clinical cancer research and practice, enabling a wider implementation of (multi‐)omics analyses for precision oncology.

## Graphical abstract

We studied a novel snap freezer and showed similar protein‐ and RNA‐based molecular profiles of biological samples including individual patient tissues as obtained with the golden standard of LN2 quenching. Importantly, this snap freezer overcomes several practical limitations of LN2 and may provide a useful tool enabling wider implementation of (multi‐)omics analyses for precision oncology.

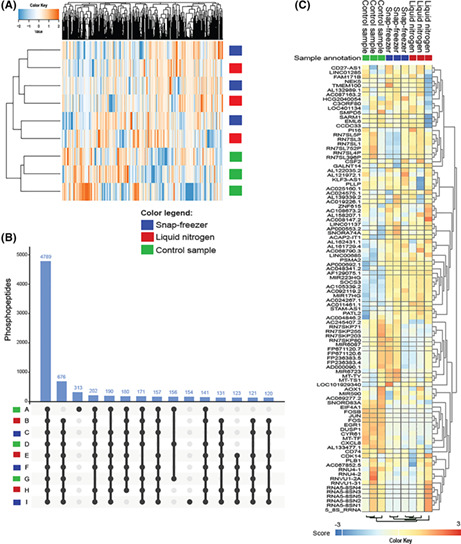

## 1 INTRODUCTION

Genomic, transcriptomic, and (phospho)proteomic profiling of tumor biopsies plays an increasingly important role in translational cancer research and precision oncology, the selection of therapy for patients with cancer based on their molecular tumor profile.^1^, ^2^, ^3^ Standardized high‐quality (cryo) preservation to most accurately harness tumor biology of assessed tissue is a prerequisite for the generation of complex DNA, RNA, and protein data.^4^, ^5^ Cryopreservation of cells and tissues demands swift cooling to sub‐freezing temperatures at which biological and enzymatic processes are slowed down or completely stopped.^6^, ^7^ Liquid nitrogen (LN_2_, −196°C or 77 K), or pre‐cooled isopentane (often −80°C) are preferred coolants to control cooling rate and prevent cryo‐artifacts in tissues, allowing their structural and biochemical preservation.^8^, ^9^, ^10^, ^11^ Tumor biopsies collected for research and precision oncology purposes are generally placed in a cryovial by trained staff and immediately immersed in LN_2_ This process is referred to as snap freezing and currently the golden standard.^12^ Snap freezing is a laborious, potentially hazardous, and not user‐friendly procedure. In addition, LN_2_ is not widely available and the use of sacrificial LN_2_ is non‐sustainable due to its energy‐intensive synthesis. There is an unmet need for a snap freezing device without these limitations that allows standardized optimal conservation of core needle biopsies or resected tissue for molecular profiling purposes.

We have previously described an electrically powered, novel snap freezer that is not reliant on LN_2_ and has adjustable cold sink temperature that will influence the cooling rate.^8^, ^13^ This apparatus consists of a cryocooler, Thermal Energy Storage Unit (TESU), and a gas handling system, which is transportable and easy to handle. Cooling occurs through a narrow gas‐gap between the cryovial and the thermal reservoir holding the vial. Recently, we showed that the cooling rate of a vial depends on the thermal properties of the vial material (e.g., aluminum, polypropylene) and on the coolant used. The cooling rate for a LN_2_‐frozen tissue biopsy in an aluminum vial was about −25°C/s.^13^

We hypothesize that this novel snap freezer will preserve quality and molecular profiles of tissue biopsies similar to and is more user‐friendly than the golden standard of LN_2_ quenching. To address this, we benchmarked the performance of the snap freezer prototype to the golden standard with regard to preservation of biology. Molecular profiles of snap frozen cell lines and human tissue biopsies were determined taking phosphoproteomic and transcriptomic profiles as a read out. The secondary aim of this study was to determine whether differences in freezing rate could influence the molecular profile of cancer cells.

## 2 METHODS

### 2.1 Cell culture, lysis, and protein digestion

Cells from chronic myeloid leukemia (CML) K562 and the colorectal cancer cell line HCT116 were cultured according to standard methods as described in Data S1.

### 2.2 Tissue biopsy collection, lysis, and protein digestion

Normal liver tissue biopsies were collected from five patients with cancer who underwent liver metastasectomy at Amsterdam UMC location VUmc in September 2019. Since the Dutch Medical Research Involving Human Subjects Act does not apply to normal adjacent tissue that is removed, this tissue could be used for research purposes; patients have the possibility to opt‐out of the use of their residual tissue for future research. For each patient and immediately after resection, six 14‐gauge core needle biopsies of adjacent normal liver tissue were taken from the resection specimen by the surgeon, placed into separate aluminum vials and snap frozen within 5 min. After below mentioned freezing procedures, biopsies were longitudinally cut in 10 μm sections (cryomicrotome, Leica CM1850) and processed to tumor lysates for mass spectrometry (MS)‐based global phopshoproteomics as described elsewhere.^14^, ^15^ Lysates were stored at −80°C.

### 2.3 Benchmarking performance snap freezer versus liquid nitrogen quenching

**FIGURE 1:**
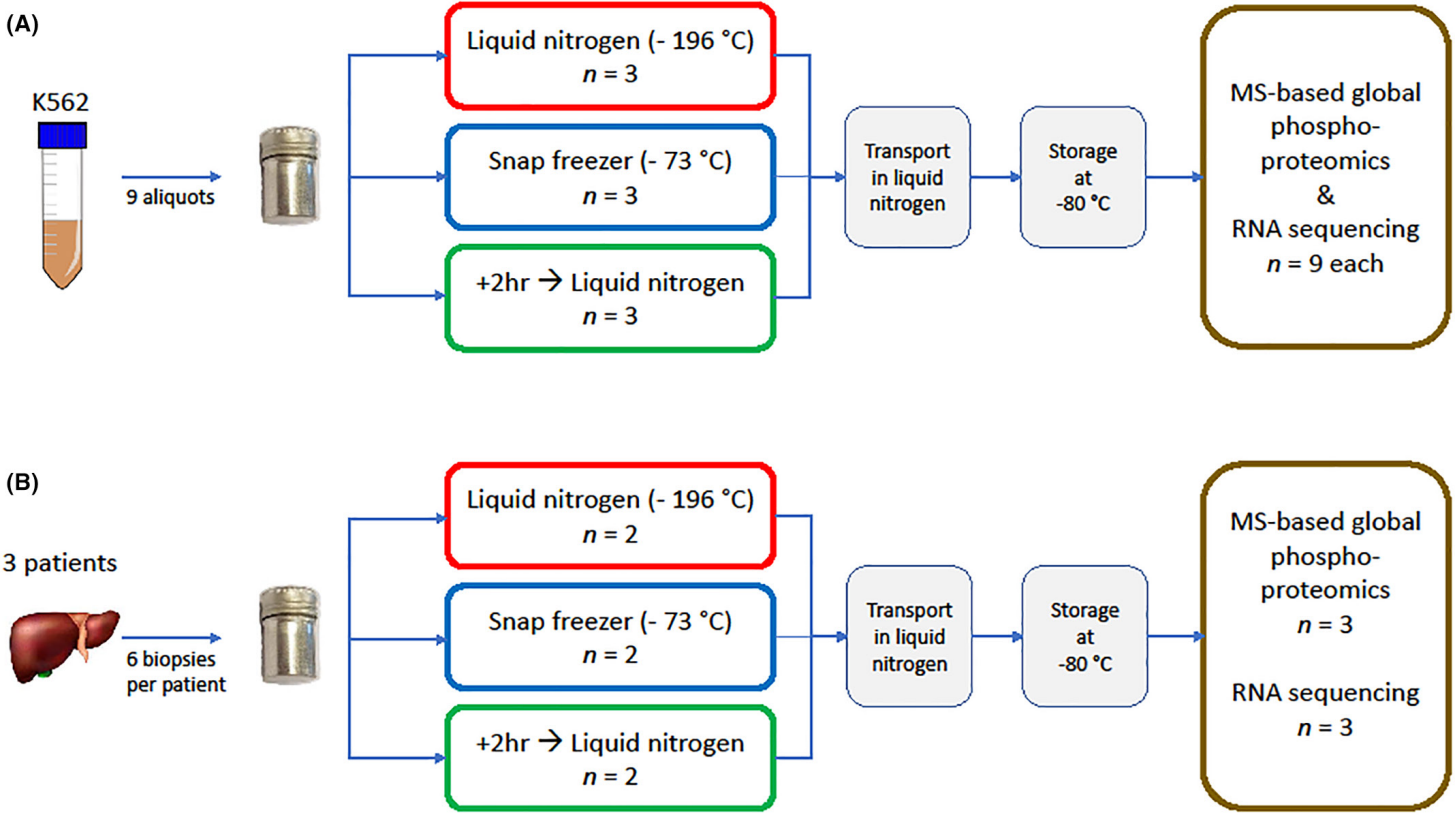
Benchmarking performance of snap freezer versus liquid nitrogen quenching.Study design to compare molecular profiles of biospecimen frozen using the snap freezer versus golden standard of liquid nitrogen quenching. (A) K562 suspension cell line samples frozen using liquid nitrogen (−196°C) versus snap freezer (−73°C). Positive control samples were kept at room temperature for 2 h before freezing in liquid nitrogen. The surplus lysate of each of the nine samples was used for RNA extraction to perform sequencing and determine RNA integrity scores. (B) Normal human liver tissue frozen using liquid nitrogen versus snap freezer (two biological replicates per condition). From each biological replicate, one sample was processed for global (TiOx) phosphoproteomics and one sample was processed for RNA extraction to perform sequencing, and determine RNA integrity scores.

Three triplicates of 5–10 mL K562 suspension cell line, each corresponding to 500 μg of protein, and 3–9 normal liver tissue biopsies per patient were snap frozen in aluminum vials by one of the following three methods: (i) cooling to −196°C by immersion in LN_2_ (golden standard), (ii) cooling to −73°C in the snap freezer, and (iii) storage at room temperature for 2 h, followed by immersion in LN_2_ to −196°C (+2 h positive control). −73°C (200 K) is in general accepted as an adequate temperature to preserve stability of biospecimens for storage.^16^, ^17^ Before start of the experiments, a vessel filled with LN_2_ was placed in the laboratory and the electrically powered snap freezer was pre‐cooled to −73°C (200 K). In each experiment, one vial was placed into the snap freezer and simultaneously another vial was immersed in LN_2_, alternatingly performed for the two tissue replicates or three cell suspension workflow replicates (Figure 1). After cooling of the vials, all vials in the experiment were transported in LN_2_ and stored in a freezer at −80°C until further use.

### 2.4 Influence of freezing rate on phosphoproteomics profile

Fifteen aliquots of 300 μL HCT116 lysate, each corresponding to 300–400 μg of protein, were placed in three types of vials with different thermal conduction properties (polypropylene, aluminum, and aluminum vials covered in paper tape) to influence their freezing rates. For each condition three vials were individually immersed in either LN_2_ or precooled isopentane for 1 min and cooled to a temperature of −196°C or −80°C, respectively, using a stainless steel vial holder. Pre‐cooled isopentane was tested as second coolant, because at room temperature, isopentane (with boiling point of 36.9°C) is in liquid phase. Therefore, no boiling occurs and the cooling rate is not subjected to the Leidenfrost effect, which is the phenomenon that a vapor layer is formed that prevents heat transfer.^18^, ^19^ The aluminum vial covered in paper tape was not immersed in isopentane, because previously published experiments have shown that this vial was not subjected to the Leidenfrost effect in LN_2_.^20^ After adequate cooling, vials were transported using a LN_2_ container and stored at −80°C until further use.

### 2.5 Phosphoproteomics: phosphopeptide enrichment, LC–MS/MS measurement, protein identification, and label‐free phosphopeptide quantification

K562 and HCT116 cell lysate aliquots and tissue lysates were reduced, alkylated, and digested as described previously.^14^ Desalted peptides were enriched for phosphopeptides using titanium oxide (TiOx) beads based using aliphatic hydroxy‐acid modified metal oxide chromatography.^21^, ^22^ Further sample preparation details are provided in Data S1. Phosphopeptides were separated by nanoLC and detected as described previously^21^, ^23^, ^24^ on a Q exactive HF mass spectrometer (Thermo Fisher). Protein identification and phosphopeptide quantification were performed as previously described.^14^ In short, LC–MS/MS spectra were searched against the Uniprot human reference proteome FASTA file (release February 2019, 42,417 entries, no fragments) using MaxQuant 1.6.4.0.^25^ (Phospho) peptide identifications were propagated across samples using the match‐between‐runs option checked. Searches were performed as previously described in detail with the label‐free quantification option selected.^24^ Phosphopeptides were quantified by their extracted ion chromatograms (“Intensity” in MaxQuant). For each sample the phosphopeptide intensities were normalized on the median intensity of all identified peptides in the sample (“normalized intensity” from the MaxQuant Evidence table). Further details are provided in the Data S1.

### 2.6 RNA extraction and integrity, RNA sequencing

Tissue: Dissection of fresh frozen biopsies was performed at −25°C in a cryotome. Twenty micrometer sections were cut and snap frozen in liquid nitrogen and stored at −80°C until RNA extraction. RNA was isolated from the tissue specimens and the surplus of K562 cell suspension samples used for the phosphoproteomics analysis, using the RNeasy Plus Mini K (Qiagen) according to the manufacturers protocol, eluted in 30 μL nuclease free water and quantified using a NanoDrop One UV–Vis Spectrophotometer (Thermo Scientific). To analyze differences in RNA integrity between samples processed in different freezing conditions, the RNA integrity number (RIN) was determined using the RNA 6000 Picochip (Bioanalyzer 2100, Agilent). The Bioanalyzer 2100 quality and quantity measures were collected from the automatically generated Bioanalyzer result reports using default settings. Next‐generation sequencing (NGS) using Illumina's TruSeq Small RNA Sample Preparation protocol and data filtering were performed as previously described.^26^ Illumina's TruSeq Small RNA Sample Preparation protocol was used for the generation of cDNA libraries. These libraries were amplified on the flow cells with Illumina's cluster station (Illumina Inc.) and sequenced using Illumina's HiSeq 2000 (Illumina Inc.). Further details are provided in Data S1.

## 3 RESULTS

### 3.1 Benchmarking of snap freezer versus liquid nitrogen quenching in molecular profiling

#### 3.1.1 Cancer cell line samples

**FIGURE 2:**
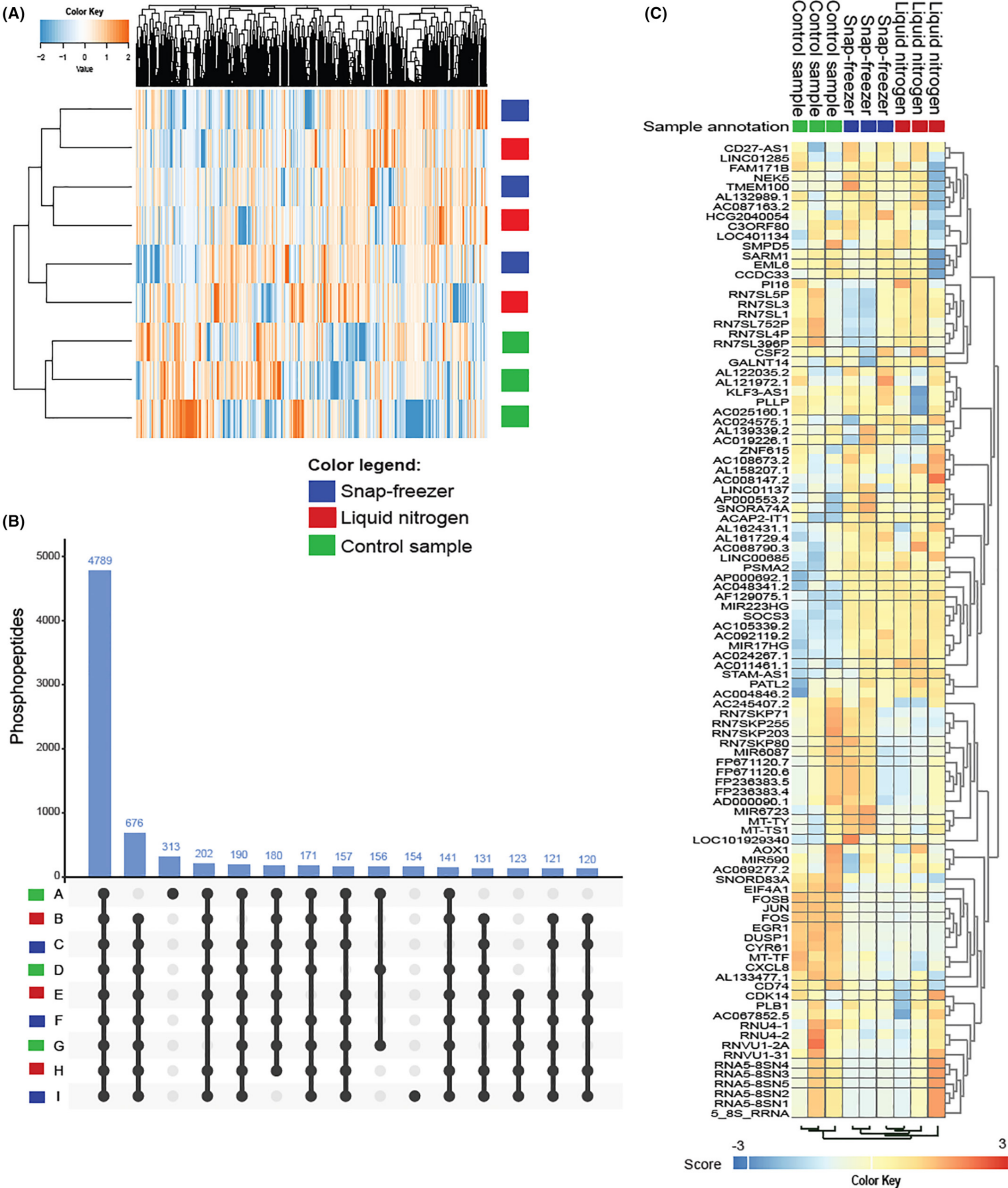
Benchmark of molecular profile preservation using K562 cancer cellsProfile preservation benchmarking using snap freezer versus liquid nitrogen. (A) Unsupervised cluster analysis of all identified phosphosites of K562 suspension aliquots does not cluster samples frozen by liquid nitrogen separately from those frozen by the snap freezer, but clearly separates the +2 h positive control samples. Color key indicates *Z*‐scores. (B) UpSet plot indicating the number of overlapping phosphopeptides shared between (subsets of) the nine K562 samples. In total, 15 out of 511 overlaps are shown, covering 51% of the data. (C) Unsupervised cluster analysis of RNA expression of 100 most varying genes does not cluster samples frozen using liquid nitrogen or the snap freezer together, but separates the +2 h positive control samples. Color key indicates *Z*‐scores.

Using a snap freezer at −73°C and the cold sink temperature of LN_2_,^13^ a comparative analysis of the phosphoproteome and transcriptome of suspension cell line K562 was performed (Figure 1A). Mass spectrometry‐based global phosphoproteomics was successfully performed on all nine (three triplicates) K562 cell suspension lysate samples. A total of 16,341 unique peptides were identified of which 14,835 (90.8%) were phosphorylated. The median number of phosphopeptides per sample was 10,357 (range 9317–10,735). The number of identified phosphopeptides did not differ significantly between both freezing methods (*p* = 0.44 by students' *t*‐test). A total of 14,812 unique phosphosites were identified (83.4% serine, 15.2% threonine, and 1.4% tyrosine), with a median of 9502 (range 8306–9871) per sample. Unsupervised cluster analysis of phosphosites did not show separation of samples processed in LN_2_ from samples processed in the snap freezer (Figure 2A). Comparison of the nine study samples with each other showed high Pearson correlations (median *r* 0.96 (range 0.92–0.98) for either direct freezing method) while the positive control samples with 2 additional hours of bench‐time did cluster separately. (Figure S1A); 4789 phosphopeptides (29% of total number of identified phosphopeptides) were shared between all samples (Figure 2B). Next, a read‐out at the transcriptomic level was used to compare LN_2_‐ versus snap freezer‐based biospecimen freezing. No significant difference was observed in RNA integrity between cell line samples processed using the two freezing methods, including the +2 h positive controls, indicating that integrity of the RNA molecules is preserved by the snap freezer(Table 1). Also, RNA molecules were shown to be stable after 2 h at room temperature (Table 1). Unsupervised cluster analysis of the 100 most variably expressed genes showed two main clusters, one smaller consisting of the three positive control samples; the second cluster was a mixed cluster of samples processed using either method (Figure 2C). The two snap freezing methods could not be distinguished based on the RNA expression profiles, even when selecting only the top 100 varying genes between the samples for clustering analysis. Again, comparison of all separate samples with each other showed very high correlation (Pearson's *r* > 0.99, Figure S1B).

**TABLE 1:** RNA integrity preservation.

Freezing method	RIN value		
	Replicate 1	Replicate 2	Replicate 3
Liquid nitrogen	9.40	9.50	9.30
Snap freezer	9.10	9.30	9.20
+2 h Control sample	9.40	9.20	9.30

*Note*: RNA integrity of cell line samples processed with different freezing methods. Aluminum vials with lysates of K562 suspension cell line were alternatingly snap‐frozen in the snap freezer or in liquid nitrogen. Three samples were left at room temperature for 2 h before freezing in liquid nitrogen as a positive control sample. RIN, RNA integrity numbers.

#### 3.1.2 Normal liver tissue biopsies

**FIGURE 3:**
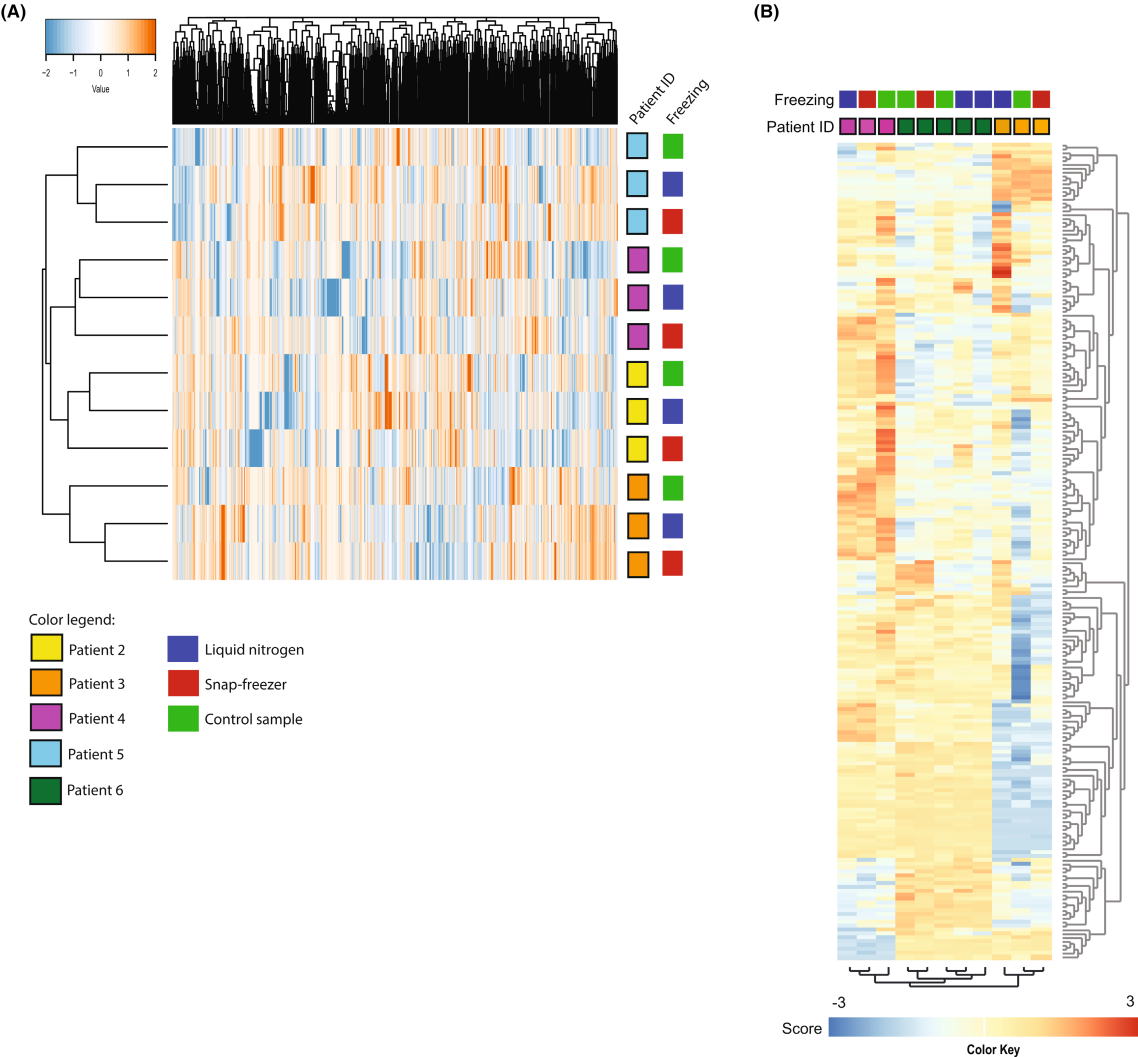
Benchmark of molecular profile preservation of normal liver biopsies from patients with cancer. Molecular profile preservation benchmark of snap freezer versus liquid nitrogen. (A) Unsupervised cluster analysis of the phosphoproteome of liver tissue samples of four individual patients shows that patient‐specific profiles can clearly be identified in samples snap frozen in the snap freezer as well as in liquid nitrogen. Color key indicates *Z*‐scores. (B) Unsupervised cluster analysis of RNA expression of 100 most variable genes shows that three individual patient profiles can be clearly identified using samples processed in both freezing methods. Color key indicates *Z*‐scores.

Characteristics and analysis details of five consecutive patients who underwent liver surgery are presented in Table S1. For patient 01 only three normal liver tissue biopsies were available (phosphoproteomics) and for patients 02, −03‐, and 04, six biopsies per patient could be evaluated for phosphoproteomics, RNA integrity analysis, and RNA sequencing. These biopsies were snap‐frozen alternatingly using the three freezing methods (Figure 1B). In total, 12 14G core needle biopsies from four patients were processed for global phosphoproteomics, with a median protein input of 500 μg per sample. A total of 15,262 unique peptides were identified, of which 10,395 (68%) were phosphorylated. The median number of phosphopeptides per sample was 6742 (range 5535–7601). A total of 9966 phosphosites were identified (86% serine, 13% threonine, and 1% tyrosine), with a median of 5794 (range 4710–6573) per sample. Unsupervised clustering of the phosphoproteome revealed clear separation of replicates from the four patients (Figure 3A). Subclustering of snap freezer‐ and LN_2_‐frozen samples, separate from the +2 h controls, was observed in two of four patients. RNA isolation was successfully performed in tissues from two of three last mentioned patients. An additional set of nine liver biopsies was obtained from a fifth patient (05, Table S1). RNA quality was insufficient in one of the biopsies, leaving 11 samples for downstream analysis. There were no significant differences in RIN values between the samples processed using the two freezing methods (*p* = 0.89 by *t*‐test). Samples that were left at room temperature for 2 h before immersion in LN_2_ had RIN values comparable to the other two freezing conditions, indicating that RNA is a stable molecule, even after a prolonged cold ischemia time (Table S1). After RNA sequencing, unsupervised cluster analysis of gene expression profiles showed a clear separation of the samples from individual patients (Figure 3B).

### 3.2 Effect of different freezing rates on phosphoproteomic profiles

**FIGURE 4:**
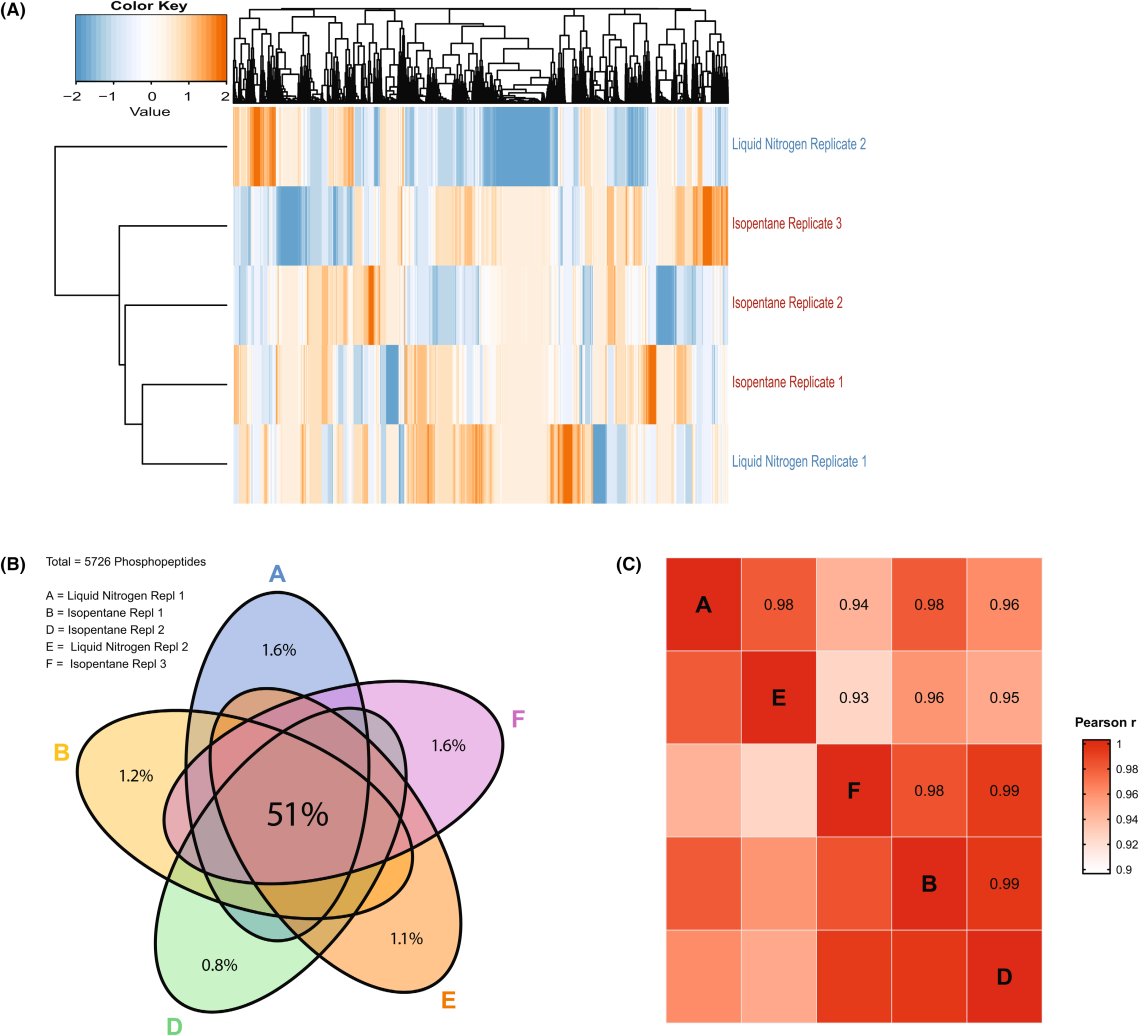
Effect of freezing rate on HCT116 phosphoproteomic profile. Effect of freezing rate on the phosphoproteome of cancer cell line HCT116. HCT116 samples were frozen in polypropylene vials in either liquid nitrogen (−196°C, in 2 s, *N* = 2; one sample was lost due to a technical error in the mass spectrometer) or in 25 s using pre‐cooled isopentane (−80°C, *N* = 3). (A) Unsupervised cluster analysis of all identified phosphopeptides does not separate samples with highest versus lowest freezing rate. (B) Venn diagram of overlapping phosphopeptides between all five samples shows reproducible phosphopeptide identification regardless of freezing rate. (C) Correlation by Pearson's *r* shows high correlation between phosphoproteomic profiles of samples frozen at high versus lower rate.

Three types of vials with different thermal conduction properties (polypropylene, aluminum, and aluminum vials covered in paper tape) and two coolants (LN_2_ and pre‐cooled isopentane) were used to determine differences in freezing rate of HCT116 cancer cell lines samples to reach −80°C^8^ (Figure S1). Polypropylene vials immersed in LN_2_ versus pre‐cooled isopentane had a mean freezing time of 2 versus 25 s, respectively, while aluminum vials without paper tape covering had freezing times of 4 s in LN_2_ and 10 s in isopentane (Table 2). To study whether changes to the phosphoproteome would be detectable in samples from vials with shortest versus longest (2 vs. 25 s) freezing time, polypropylene vials frozen in LN_2_ versus isopentane were selected for molecular analysis by MS‐based global phosphoproteomics. This was successfully performed in five out of six samples. One LN_2_‐cooled sample was lost due to a technical error in the mass spectrometer. A total of 8597 unique peptides were identified of which 5726 (66.6%) were phosphorylated, reflecting adequate enrichment for phosphopeptides. The median number of identified phosphopeptides per sample (500 μg protein input/sample) was 4668 (range 4035–4780). A total of 5643 unique phosphosites were identified (phosphorylated in 87% at serine residues, 12% threonine and 1% tyrosine), with a median of 4127 (range 3765–4251) phosphosites per sample. Unsupervised clustering of the global phosphoproteome did not separate HCT116 samples frozen in polypropylene vials of 2 versus 25 s freezing rates (Figure 4A). Fifty‐one percent of all identified phosphopeptides were present in all five samples and only ≤1.6% were uniquely identified in one of the samples; 47%–48% of identified phosphopeptides per sample were present in at least one other sample (Figure 4B). The overlap between workflow replicates (47% for LN_2_ and 51% for isopentane, data not shown) was comparable to the overlap between the different conditions (51% as per the Venn diagram in Figure 4B). The correlation between all samples was high (Pearson's *r* 0.93–0.99, Figure 4C).

**TABLE 2:** Freezing rates depending on vial type and coolant.

Vial type	Liquid nitrogen	Precooled isopentane
Aluminum	4 s	10 s
Polypropylene	2 s	25 s
Aluminum covered in paper tape	<2 s	N/A

*Note*: Three different types of vials containing HCT116 lysate were immersed in either liquid nitrogen (−196°C) or pre‐cooled isopentane (−80°C; Figure 2). The time in seconds (s) elapsed from the point of room temperature until the vials reached a temperature of −80°C. Three technical replicates per freezing condition were used.

## 4 DISCUSSION

Snap freezing of core needle biopsies by quenching in liquid nitrogen (LN_2_) is the golden standard to preserve tumor biology and allow profiling for precision medicine purposes at the DNA, RNA, and (phospho)protein level, but the use of LN_2_ has several disadvantages. We have previously developed a LN_2_‐independent, electrically powered and mobile snap freezer with adjustable cold sink temperature.^13^ Comparing the novel snap freezer with the golden standard of LN_2_ quenching, we here show that MS‐based phosphoproteomic and transcriptomic profiles of cancer cell line K562 and human liver biopsies are preserved (Figures 2 and 3). Phosphoproteome differences between individual patients were larger than potential differences induced by either freezing method (Figure 3A). Gene expression profiling by RNA sequencing corroborated these findings (Figure 3B). These findings are important, because MS‐based phosphoproteomics and RNA sequencing profiles are sensitive to variation induced by differences in pre‐analytical handling that impact tissue integrity. Ultimately, such variations would hamper extrapolation and implementation of research findings to the general patient population.^27^, ^28^ In particular, cold ischemia time can alter the (phospho)proteome and transcriptome.^29^, ^30^, ^31^ While DNA in tumor tissue remains stable after 1 h of cold ischemia time,^32^, ^33^ earlier studies describe multiple examples of altered protein and mRNA expression within 15–30 min and phosphorylation as early as after 5 min of cold ischemia time.^34^, ^35^, ^36^, ^37^ Remarkably, MS‐based phosphotyrosine (pY)‐phosphoproteomic profiles from acute myeloid leukemia samples were recently shown to remain relatively stable after a 4‐h delay of sample processing.^38^ These results may indicate that the impact of pre‐analytical variation may differ for hematological specimens versus solid tumor biopsies, but need further confirmation. In general, standard methods resulting in reliable results with minimal variation are prerequisites for application in precision oncology. Here, we found that the novel snap freezer is fulfilling this requirement by showing that molecular profiles of cell lines and individual patients' biopsies were maintained.

In addition, the effect of freezing rate differences on the phosphoproteomic profile of a cancer cell line was evaluated. Freezing rates that are too low will damage the cell membrane, likely due to increased solute concentration caused by volume reduction of liquid surrounding the cells,^39^ while ultra‐rapid cooling may lead to damage through devitrification and ice crystal formation upon storage including the Leidenfrost phenomenon.^20^ We here found that differences in freezing rate up to 23 s to a goal temperature of −73°C did not induce significant changes in phosphoproteomic profiles (Figure 4) indicating that a freezing rate faster than achieved with the snap freezer and with LN_2_ is unnecessary. Increasing the freezing rate by overcoming the Leidenfrost effect will not further improve preservation of the molecular profile of a biological sample. Together, these results imply that this snap freezer is of valid use in clinical setting, eliminating the need for harmful coolants and preventing technical and practical challenges of LN_2_ for cryopreservation. Alternative snap freezing solutions have been developed to circumvent the limitations of liquid nitrogen, but each of them has limitations in terms of mobility or cooling performance.^40^, ^41^

As in vivo profiling of (tumor) tissue is impossible, one cannot perform molecular profiling without potentially inducing any procedure‐related effect. It is impossible to determine which of both snap freezing methods preserves in vivo profiles best. Cancer cell samples left at room temperature for 2 h prior to snap freezing were used as a control to show that profiles do change in time. However, when optimal sampling of biospecimens is clinically implemented, no significant differences in molecular profiles are expected based on the freezing rate experiments as described here. This study was designed to compare technical replicates. Although the included clinical sample size was small, results were consistent throughout all comparisons of both phosphoproteomic and RNA sequencing analyses.

In conclusion, the novel snap freezer prototype identifies similar protein‐ and RNA‐based molecular profiles of biological samples including individual patient tissues as obtained with the golden standard of LN_2_ quenching. Importantly, this snap freezer overcomes several practical limitations of LN_2_ and may provide a useful tool enabling wider implementation of (multi‐)omics analyses for precision oncology. Feasibility and usability for snap freezing tumor biopsies in the context of a (precision oncology) clinical trial or the routine clinical setting should be assessed as the next critical step toward its implementation and commercial development.

## AUTHOR CONTRIBUTIONS

**Hanneke van der Wijngaart:** Conceptualization (equal); data curation (equal); formal analysis (equal); investigation (equal); methodology (equal); project administration (equal); validation (equal); visualization (equal); writing – original draft (equal). **Sahil Jagga:** Conceptualization (equal); data curation (equal); formal analysis (equal); investigation (equal); methodology (equal); validation (equal); visualization (equal); writing – review and editing (equal). **Henk Dekker:** Data curation (equal); formal analysis (equal); investigation (equal); methodology (equal); visualization (equal); writing – review and editing (equal). **Richard de Goeij:** Conceptualization (equal); formal analysis (equal); investigation (equal); methodology (equal); supervision (equal); validation (equal); writing – review and editing (equal). **Sander R. Piersma:** Conceptualization (equal); data curation (equal); formal analysis (equal); investigation (equal); methodology (equal); validation (equal); visualization (equal); writing – review and editing (equal). **Thang V. Pham:** Conceptualization (equal); formal analysis (equal); investigation (equal); methodology (equal); validation (equal); visualization (equal); writing – review and editing (equal). **Jaco C. Knol:** Conceptualization (equal); formal analysis (equal); investigation (equal); methodology (equal); validation (equal); writing – review and editing (equal). **Babs M. Zonderhuis:** Conceptualization (equal); investigation (equal); methodology (equal); writing – review and editing (equal). **Harry J. Holland:** Conceptualization (equal); investigation (equal); methodology (equal); validation (equal); writing – review and editing (equal). **Connie R. Jiménez:** Conceptualization (equal); investigation (equal); methodology (equal); supervision (equal); validation (equal); visualization (equal); writing – review and editing (equal). **Henk M. W. Verheul:** Conceptualization (equal); formal analysis (equal); funding acquisition (equal); investigation (equal); methodology (equal); supervision (equal); validation (equal); writing – original draft (equal); writing – review and editing (equal). **Srinivas Vanapalli:** Conceptualization (equal); data curation (equal); formal analysis (equal); funding acquisition (equal); investigation (equal); methodology (equal); project administration (equal); resources (equal); software (equal); supervision (equal); validation (equal); visualization (equal); writing – original draft (equal); writing – review and editing (equal). **Mariette Labots:** Conceptualization (equal); data curation (equal); formal analysis (equal); funding acquisition (equal); investigation (equal); methodology (equal); project administration (equal); resources (equal); software (equal); supervision (equal); validation (equal); visualization (equal); writing – original draft (equal); writing – review and editing (equal).

## Supporting information


Figure S1

Figure S2

Table S1

Data S1

## Data Availability

The data that support the findings of this study are available from the corresponding author upon reasonable request.
