# Supplementary figures and images for: Advancing wide implementation of precision oncology: A liquid nitrogen‐free snap freezer preserves molecular profiles of biological samples
Source: Cancer Med. 2023 Mar 14;12(9):10979–89. doi: 10.1002/cam4.5781 (PMC10225239; doi:10.1002/cam4.5781)

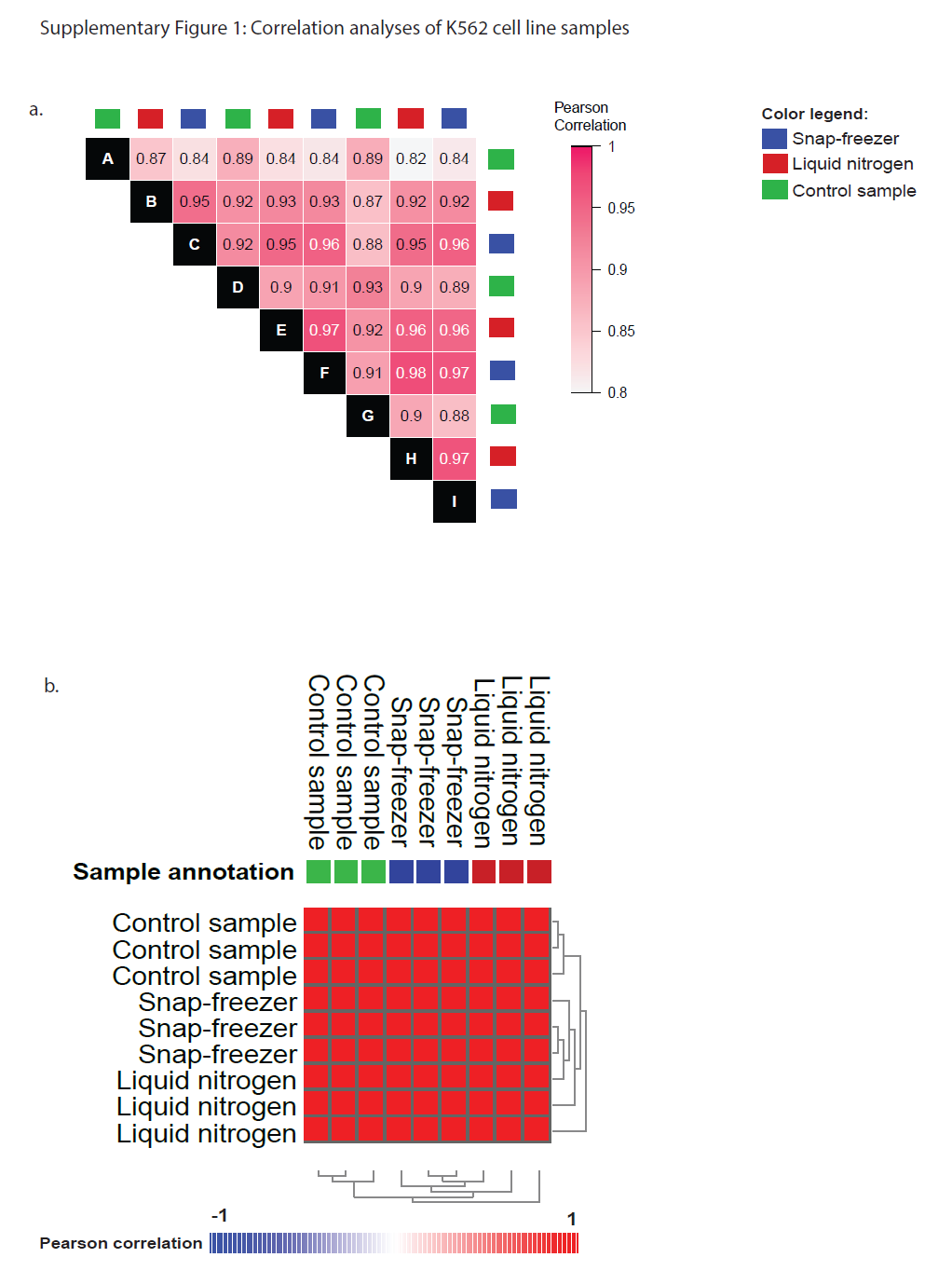

Supplement: Supplementary file 1 — Figure S1 [file CAM4-12-10979-s002.docx]

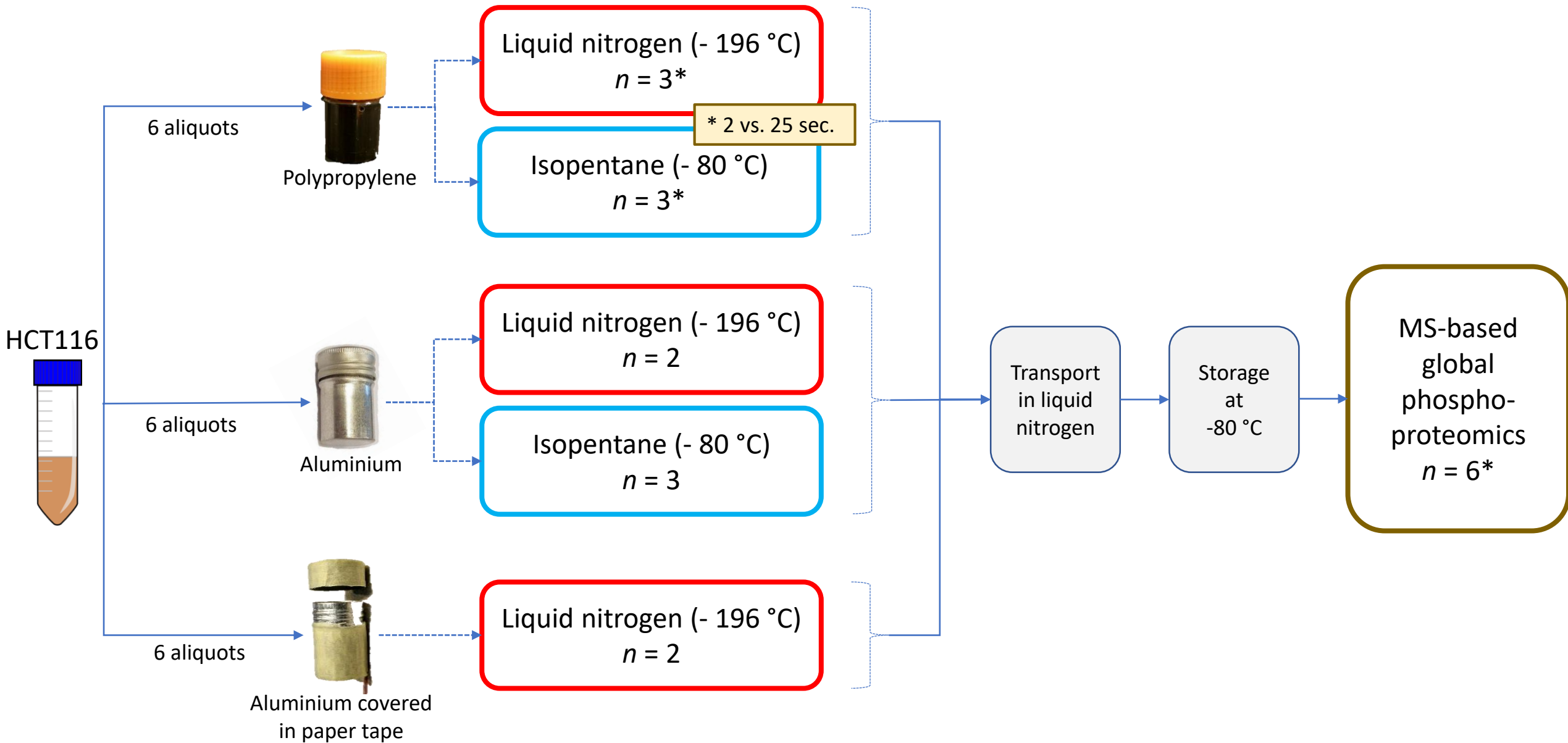

Supplement: Supplementary file 2 — Figure S2 [file CAM4-12-10979-s004.pdf]
