# Supplementary material for: Advancing wide implementation of precision oncology: A liquid nitrogen‐free snap freezer preserves molecular profiles of biological samples
Source: Cancer Med. 2023 Mar 14;12(9):10979–89. doi: 10.1002/cam4.5781 (PMC10225239; doi:10.1002/cam4.5781)
Supplement: Supplementary file 4 — Data S1 [file CAM4-12-10979-s003.docx]

Supplementary Materials to

# Advancing wide implementation of precision oncology: a liquid nitrogen-free snap freezer preserves molecular profiles of biological samples

## Authors:

H. van der Wijngaart^1^, S. Jagga^2^, H. Dekker^1,4^, R. de Goeij^1,4^, S.R. Piersma^1,4^, T.V. Pham^1,4^, J.C. Knol^1,4^, B. Zonderhuis^3^, H.J. Holland^2^, C.R. Jiménez^1,4^, H.M.W. Verheul^5^, S. Vanapalli^2^, M. Labots^1,4^

1. Amsterdam UMC location Vrije Universiteit Amsterdam, Department of Medical Oncology, De Boelelaan 1117, Amsterdam, the Netherlands
2. Applied Thermal Sciences, Faculty of Science and Technology, University of Twente, 7500 AE Enschede, The Netherlands
3. Amsterdam UMC location Vrije Universiteit Amsterdam, Department of Surgery, De Boelelaan 1117, Amsterdam, the Netherlands
4. Cancer Center Amsterdam, Imaging and Biomarkers, Amsterdam, the Netherlands
5. Department of Medical Oncology, Erasmus University Medical Center, The Netherlands

***Supplementary Materials***

- Supplementary Methods
- Supplementary Table 1
- Legends Supplementary Figure 1 and 2

**Supplementary Methods**

*Patient samples*

This study received approval from the Amsterdam UMC Biobank under study number BUP2019-12. Regular diagnostic procedures were not hindered by the collection of the study biopsies.

*Cell lines*

K562 chronic myeloid leukemia (CML) cells were obtained from the ATCC and cultured in DMEM medium supplemented with 10% FBS (Biowest, France). Cells were maintained at 37 °C and expanded in a T175 culture flaks. Nine aliquots of 10 ml cell suspension (exponential growth phase) were transferred into a 50 ml tube and centrifuged for 2 minutes at 300g and the supernatant was removed. Cells were washed twice in phosphate-buffered saline (PBS) and centrifuged for 2 minutes at 300g before being resuspended in 1 ml PBS and transferred in the aluminum vial. The vial was placed into a 50 ml tube and centrifuged for 1 minute at 300g rpm, after which PBS was removed and the pellet of cells remained on the bottom of the vial. The vials subsequently entered their respective freezing procedures, see below, and were stored at -80 °C until further processing.

Cells from the colorectal cancer cell line HCT116 were cultured in biological triplicates in DMEM medium (Lonza Biowhittaker, Verviers, Belgium) containing 10% fetal bovine serum, 2 mM glutamine, 100 IU/ml sodium penicillin and 100 µg/ml streptomycin. Cells were lysed in lysis buffer containing 9 M urea, 20 mM HEPES pH 8.0, 1 mM Na_3_VO_4_ (orthovanadate), 2.4 mM Na_4_P_2_O_7_ (pyrophosphate), and 1 mM Na_2_C_3_H_7_PO_6_ (β-glycerophosphate) by scraping and subsequent sonication. After lysis, protein concentration was determined using the BCA method (ThermoPierce, Rockford, IL). Cell lysate was reduced in 4 mM dithiotreitol (DTT) for 20 minutes at 60 °C, cooled to room temperature and alkylated in 10 mM iodoacetamide for 15 minutes in the dark. Next, the cell lysate was diluted to 2 M urea using 20 mM HEPES buffer pH 8.0 and digested overnight with trypsin (10 µg/mg protein) at 37 °C. Digestion was stopped in 0.1% trifluoroacetic acid (TFA).

*Phosphoproteomics*

*Phosphopeptide enrichment and LC-MS/MS measurement for*

HCT116 cell lysate aliquots of 300-400 µg protein, K562 cell lysate aliquots of 500 µg protein and tissue lysates were reduced, alkylated and digested as described previously^1^. Desalted peptides were enriched for phosphopeptides using titanium oxide (TiOx) beads based using aliphatic hydroxyl-acid modified metal oxide chromatography^2,3^. In brief, 500 µg desalted peptides (1 µg/µl in 80% ACN, 0.1% TFA) were mixed with 500 µl washing buffer (80% ACN, 0.1% TFA containing 300 mg/ml lactic acid) and applied to 2.5 mg TiOx beads (GL sciences, 10 µm) packed in a 200 µl STAGE tip containing a 16G empore C8 membrane plug (3M, St Paul, MN). The STAGE tip was washed with 200 µl washing buffer followed by 200 µl 80% ACN, 0.1% TFA. Phosphopeptides were eluted in two steps in 50 µl 0.5% and 5% piperidine (Fisher Scientific) and were quenched in 100 µl 20% H_3_PO_4_. All steps were performed by centrifugation (1500 x g, 4 min). Phosphopeptides were desalted using a 200 µl STAGE tip containing a 16G empore SDB-XC membrane plug (3 M, St Paul, MN) using the same solvents as used for the Seppak cartridge (20 µl, 100 rpm, 1 min). Desalted phosphopeptides were dried in a vacuum centrifuge and redissolved in 20 µl 4% ACN, 0.5% TFA; 17 µl was injected on the column. Phosphopeptides were separated by nanoLC and detected as described elsewhere ^2,4,5^ on a Q exactive HF mass spectrometer (Thermo Fisher, Bremen, Germany).

*Protein identification*

LC-MS/MS spectra were searched against the Uniprot human reference proteome FASTA file (release February 2019, 42417 entries, no fragments) using MaxQuant 1.6.4.0^6^. Enzyme specificity was set to trypsin and up to two missed cleavages were allowed. Cysteine carboxamidomethylation (Cys, +57.021464 Da) was treated as fixed modification and serine, threonine and tyrosine phosphorylation (+79.966330 Da), methionine oxidation (Met, +15.994915 Da) and N-terminal acetylation (N-terminal, +42.010565 Da) as variable modifications. Peptide precursor ions were searched with a maximum mass deviation of 4.5 ppm and fragment ions with a maximum mass deviation of 20 ppm. Peptide, protein and site identifications were filtered at a false discovery rate (FDR) of 1% using the decoy database strategy. The minimal peptide length was 7 amino acids and the minimum Andromeda score for modified peptides was 40, with the corresponding minimum delta score set at 17^7^. Proteins that could not be differentiated based on MS/MS spectra alone were grouped into protein groups (default MaxQuant settings). (Phospho)peptide identifications were propagated across samples using the match-between-runs option checked. Searches were performed with the label-free quantification option selected. (Phospho)peptide identifications were propagated across samples using the match-between-runs option checked. Searches were performed with the label-free quantification option selected.

*Label-free phosphopeptide quantification*

Phosphopeptides were quantified by their extracted ion chromatograms (‘Intensity’ in MaxQuant). For each sample the phosphopeptide intensities were normalized on the median intensity of all identified peptides in the sample (‘normalized intensity’ from the MaxQuant Evidence table). Normalization and statistical testing were performed in R. Fold-change and p values were calculated from replicates using a two-tailed Student’s t-test; phosphopeptides were considered significantly differential at p < 0.05. The match-between-runs option in MaxQuant was used. Missing values were excluded from subsequent statistical analysis. Quantitative values from replicates were averaged prior to biological group comparisons. The t-test requires at least two quantitative values in each group. P-values were not corrected for multiple hypothesis testing. Cluster analysis of differential phosphopeptides was performed using hierarchical clustering in R and repeated for the top10 and 20% most variable peptides. Phosphopeptide intensities were normalized to zero mean and unit variance for each phosphopeptide. Subsequently, the Euclidean distance measure was used for phosphopeptide clustering. For sample clustering metrics, the (1-Pearson correlation) distance and the Ward linkage were used.

*RNA sequencing*

Next generation sequencing (NGS) using Illumina’s TruSeq Small RNA Sample Preparation protocol and data filtering were performed as previously described^8^. Illumina’s TruSeq Small RNA Sample Preparation protocol was used for the generation of cDNA libraries. These libraries were amplified on the flow cells with Illumina’s cluster station (Illumina Inc, San Diego, CA, USA) and sequenced using Illumina’s HiSeq 2000 (Illumina Inc, San Diego, CA, USA). Obtained sequence reads were first quality trimmed, resulting in a >99.9% probability of a correctly identified base of the remaining nucleotides. Secondly, the reads were clipped for adaptor sequences. Thirdly, reads with identical sequences were compiled and counted, resulting in only unique sequences. Finally, each unique sequence was mapped to the reference genome (browser hg19) and only those alignments of at least 18 nucleotides and a maximum of 2 mismatches were retained. Data was visualized on the R2 genomics analysis and visualization platform (<http://r2.amc.nl/>) and the R2 program was used to generate unsupervised clustering heatmaps using the View Geneset option with 100 most varying genes between the groups as found with the TopLister option, with and log2_z-score transformation settings, as well as sample correlation analyses using the Sample Correlation Map (SCM) option with data as input and log2 transformation setting. Genes with Benjamini and Hochberg p-value ≤ 0.01 were considered differentially expressed.

**Supplementary Table 1**

| **Patient ID** | **Age (years)** | **Gender** | **Primary tumor type** | **Indication for surgery** | **Biopsies** | **Freezing method** | **Processed for** | **Number of phosphopeptides** | **Total number of peptides** | **RNA Integrity number** | **Remarks** |
| --- | --- | --- | --- | --- | --- | --- | --- | --- | --- | --- | --- |
| 1 | 71 | M | Cholangio-  Carcinoma | Resection of primary tumor | 1 | Snap-Freezer | TIOX | 6054 | 9225 |  |  |
|  |  |  |  |  | 2 | LN2 | TIOX | 6506 | 9744 |  |  |
|  |  |  |  |  | 3 | Control sample | TIOX | 7067 | 10346 |  |  |
|  |  |  |  |  |  |  |  |  |  |  |  |
| 2 | 60 | F | Colorectal  cancer | Hemihepatectomy for liver metastases | 1 | Snap-freezer | TIOX | 7355 | 11053 |  |  |
|  |  |  |  |  | 2 | LN2 | TIOX | 7233 | 10821 |  |  |
|  |  |  |  |  | 3 | Control sample | TIOX | 7060 | 10690 |  |  |
|  |  |  |  |  | 4 | Snap-freezer | RNA |  |  | 9.50 |  |
|  |  |  |  |  | 5 | LN2 | RNA |  |  | 8.20 |  |
|  |  |  |  |  | 6 | Control sample | RNA |  |  | 9.30 |  |
|  |  |  |  |  |  |  |  |  |  |  |  |
| 3 | 76 | M | Colorectal  cancer | Hemihepatectomy for liver metastases | 1 | Snap-freezer | TIOX | 6456 | 10036 |  |  |
|  |  |  |  |  | 2 | LN2 | TIOX | 5535 | 8855 |  |  |
|  |  |  |  |  | 3 | Control sample | TIOX | 6003 | 9513 |  |  |
|  |  |  |  |  | 4 | Snap-freezer | RNA |  |  | 6.90 |  |
|  |  |  |  |  | 5 | LN2 | RNA |  |  | 7.80 |  |
|  |  |  |  |  | 6 | Control sample | RNA |  |  | 6.70 |  |
|  |  |  |  |  |  |  |  |  |  |  |  |
| 4 | 81 | M | Hepatocellular carcinoma | Resection of primary tumor | 1 | Snap-freezer | TIOX | 6978 | 10121 |  |  |
|  |  |  |  |  | 2 | LN2 | TIOX | 7601 | 11035 |  |  |
|  |  |  |  |  | 3 | Control sample | TIOX | 6293 | 9338 |  |  |
|  |  |  |  |  | 4 | Snap-freezer | RNA |  |  | n/a | RNA isolation failed |
|  |  |  |  |  | 5 | LN2 | RNA |  |  | n/a | RNA isolation failed |
|  |  |  |  |  | 6 | Control sample | RNA |  |  | n/a | RNA isolation failed |
|  |  |  |  |  |  |  |  |  |  |  |  |
| 5 | 55 | M | Colorectal  cancer | Hemihepatectomy for liver metastases | 1 | Snap-freezer | RNA |  |  | 7.10 |  |
|  |  |  |  |  | 2 | LN2 | RNA |  |  | 6.60 |  |
|  |  |  |  |  | 3 | Control sample | RNA |  |  | 8.30 |  |
|  |  |  |  |  | 4 | Snap-freezer | RNA |  |  | 6.90 |  |
|  |  |  |  |  | 5 | LN2 | RNA |  |  | n/a | RNA isolation failed |
|  |  |  |  |  | 6 | Control sample | RNA |  |  | 6.20 |  |
|  |  |  |  |  | 7 | Snap-freezer | n/a |  |  |  | Sample not processed |
|  |  |  |  |  | 8 | LN2 | n/a |  |  |  | Sample not processed |
|  |  |  |  |  | 9 | Control sample | n/a |  |  |  | Sample not processed |

### **Supplementary Table 1: Patient characteristics and sample processing**

14 Gauge core needle biopsies were obtained from normal liver tissue in resection specimens from five patients undergoing liver surgery in our hospital. Patient details, as well as the individual biopsies acquired from each patient and the type of analysis performed are presented. The freezing condition “control sample” indicates the positive control that was left at room temperature for two hours, before freezing in liquid nitrogen. For patient 01 only 3 normal liver tissue biopsies were available (phosphoproteomics) and for patients 02, -03- and -04, 6 biopsies per patient could be evaluated for phosphoproteomics, RNA integrity analysis and RNA sequencing. RNA isolation from three biopsies from patient 04 and one biopsy from patient 05 failed.

Abbreviations: LN2 = liquid nitrogen, n/a = not applicable, RIN = RNA integrity number, RNA = ribonucleic acid, TIOX = Titanium Oxide, an enrichment method for label-free quantitative phosphoproteomics.

**Supplementary Figures**

### **Supplementary Figure 1: Correlation analyses of K562 cell line samples**

1. Sample correlation map indicating Pearson’s r based on phosphoproteomics data of nine K562 samples. The median *r* was 0.96 (range 0.92-0.98) for either direct freezing method.
2. Sample correlation map indicating Pearson’s r based on RNA sequencing data of nine K562 samples. All samples are highly correlated, with Pearson’s r > 0.99.

### **Supplementary Figure 2: Influence of freezing rate on phosphoproteomic profiles**

*Experimental design of the comparison of the effect of 5 different freezing rates, achieved using 3 different types of vials in 2 different coolants, on the phosphoproteome using cancer cell line HCT116.* Aliquots of 300 µl HCT116 lysate, each corresponding to 300-400 µg of protein, were placed in three types of vials with different thermal conduction properties (polypropylene, aluminum and aluminum vials covered in paper tape) to influence their freezing rates. For each condition three vials were individually immersed in either LN_2_ or precooled isopentane for 1 minute and cooled to a temperature of -196 °C or -80 °C, respectively, using a stainless steel vial holder. The time to reach the goal temperature of -80 °C was registered.

**References**

1. Labots M, Pham TV, Honeywell RJ, et al: Kinase Inhibitor Treatment of Patients with Advanced Cancer Results in High Tumor Drug Concentrations and in Specific Alterations of the Tumor Phosphoproteome. Cancers (Basel) 12, 2020

2. Piersma SR, Knol JC, de Reus I, et al: Feasibility of label-free phosphoproteomics and application to base-line signaling of colorectal cancer cell lines. J Proteomics 127:247-58, 2015

3. Sugiyama N, Masuda T, Shinoda K, et al: Phosphopeptide enrichment by aliphatic hydroxy acid-modified metal oxide chromatography for nano-LC-MS/MS in proteomics applications. Mol Cell Proteomics 6:1103-9, 2007

4. van der Mijn JC, Labots M, Piersma SR, et al: Evaluation of different phospho-tyrosine antibodies for label-free phosphoproteomics. Journal of Proteomics 127:259-263, 2015

5. Beekhof R, van Alphen C, Henneman AA, et al: INKA, an integrative data analysis pipeline for phosphoproteomic inference of active kinases (vol 15, e8250, 2019). Molecular Systems Biology 15, 2019

6. Cox J, Mann M: MaxQuant enables high peptide identification rates, individualized p.p.b.-range mass accuracies and proteome-wide protein quantification. Nature Biotechnology 26:1367-1372, 2008

7. Marx H, Lemeer S, Schliep JE, et al: A large synthetic peptide and phosphopeptide reference library for mass spectrometry-based proteomics. Nature Biotechnology 31:557-+, 2013

8. Neerincx M, Sie DLS, van de Wiel MA, et al: MiR expression profiles of paired primary colorectal cancer and metastases by next-generation sequencing. Oncogenesis 4, 2015
